# Technical Feasibility of Subaxial Cervical Pedicle Screws for Distal Anchoring of Occipitocervical Fixation Constructs in the Mid-Cervical Spine: Early Clinical Experience

**DOI:** 10.7759/cureus.25964

**Published:** 2022-06-15

**Authors:** Michael A Bohl, S. Harrison Farber, U. Kumar Kakarla, Zaman Mirzadeh, Jay D Turner

**Affiliations:** 1 Department of Neurosurgery, Barrow Neurological Institute, Phoenix, USA

**Keywords:** subaxial cervical spine, occipitocervical fusion, freehand technique, cervical pedicle screws, case series

## Abstract

Occipitocervical fixation and fusion (OCF) is performed for patients who have destabilizing traumatic injuries or pathologies affecting the complex bony and ligamentous structures of the occipitoatlantal and atlantoaxial joint structures. Distal fixation failure and pseudoarthrosis are known risks of these constructs, especially for those constructs ending in the mid-cervical spine. We present the technical feasibility of using cervical pedicle screws (CPSs) as distal fixation anchors to strengthen OCF constructs ending in the mid-cervical spine and present a case series describing our early clinical experience with this technique. We used a freehand technique to place subaxial pedicle screws in the mid-cervical spine as the distal fixation point in OCF constructs. This technique involves performing a laminotomy to provide direct visualization of the pedicle borders to safely guide freehand pedicle screw placement. Our early clinical experience with this technique is presented. Three patients received OCF constructs ending in the mid-cervical subaxial spine between C3 and C6. CPSs were placed at the distal vertebra in each construct. Stable instrumentation and arthrodesis were confirmed postoperatively in all patients. This freehand technique uses direct visualization of the pedicle to aid in safe and accurate subaxial pedicle screw placement. CPS placement is clinically feasible and increases the robustness of OCF constructs in appropriately selected patients. Larger case series are needed to further validate the safety and effectiveness of this technique.

## Introduction

Occipitocervical fixation and fusion (OCF) is indicated in patients who have destabilizing injuries or pathologies affecting the complex bony and ligamentous structures that comprise the occipitoatlantal and atlantoaxial joint structures. Previous OCF techniques used onlay bone graft, in which morselized bone is placed within the surgical cavity for noninstrumented fusion and arthrodesis, and external halo or cast fixation [1,2]. External fixation was initially replaced with internal fixation techniques, such as sublaminar or spinous process wiring, and later replaced with more modern fixation techniques, such as occipital plate and cervical screw and rod constructs [3-7]. Improved internal fixation techniques have been progressively adopted over recent decades because they are believed to reduce the risk of pseudoarthrosis and instrumentation failure. Long-segment occipitocervical fixation constructs, or those placed to treat highly destabilizing injuries, are still associated with a significant risk of distal fixation failure and pseudoarthrosis [8].

It is well known that long-segment spinal fixation constructs place excessive force on distal fixation anchors because proximal construct loads are amplified by a longer lever arm. A widely accepted surgical strategy for protecting distal fixation points in thoracolumbar reconstructions is the placement of large pelvic bolts that help shield sacral fixation screws. Although OCFs are not typically as long as thoracolumbar reconstructions, distal fixation anchor failure is similarly challenging because the full load of the cranium is transmitted to smaller distal fixation anchors, such as subaxial lateral mass screws in the mid-cervical spine. Abumi et al. [8] addressed this problem more than 20 years ago in their case series of 26 patients who underwent OCF with cervical pedicle screws (CPSs) as their distal fixation anchors. Nine of these patients had CPSs placed between C3 and C6 using fluoroscopic guidance. There were no incidences of vertebral artery or nerve root injury and no reports of distal fixation failure in their series. Despite these promising early results, no subsequent studies have been published in the last 20 years on this technique. Many have since reported that subaxial CPSs are unsafe, whereas others have published extensively on the feasibility and safety of learning and clinically implementing this technique [9-19]. The purpose of this case series is to highlight the potential utility and technical feasibility of using CPSs as distal fixation anchors for OCF constructs ending in the mid-cervical spine and to describe a technique for subaxial CPS placement that minimizes the risk of neurovascular injury.

## Case presentation

The CPS placement technique is described below, and a retrospective case series describing our early clinical experience with this technique from a single academic institution is presented. Consecutive patients in whom this technique was deemed appropriate are presented. A waiver was obtained from our Institutional Review Board for this retrospective series because no protected health information is presented.

From February 2017 to February 2020, three patients underwent OCF with a distal fixation point between C3 and C6 during operations performed by the senior authors of the study. CPSs were placed at the distal fixation point in all three patients using the freehand technique described below. All patients gave consent before undergoing the procedure.

Freehand technique for subaxial CPS placement

**Figure 1:**
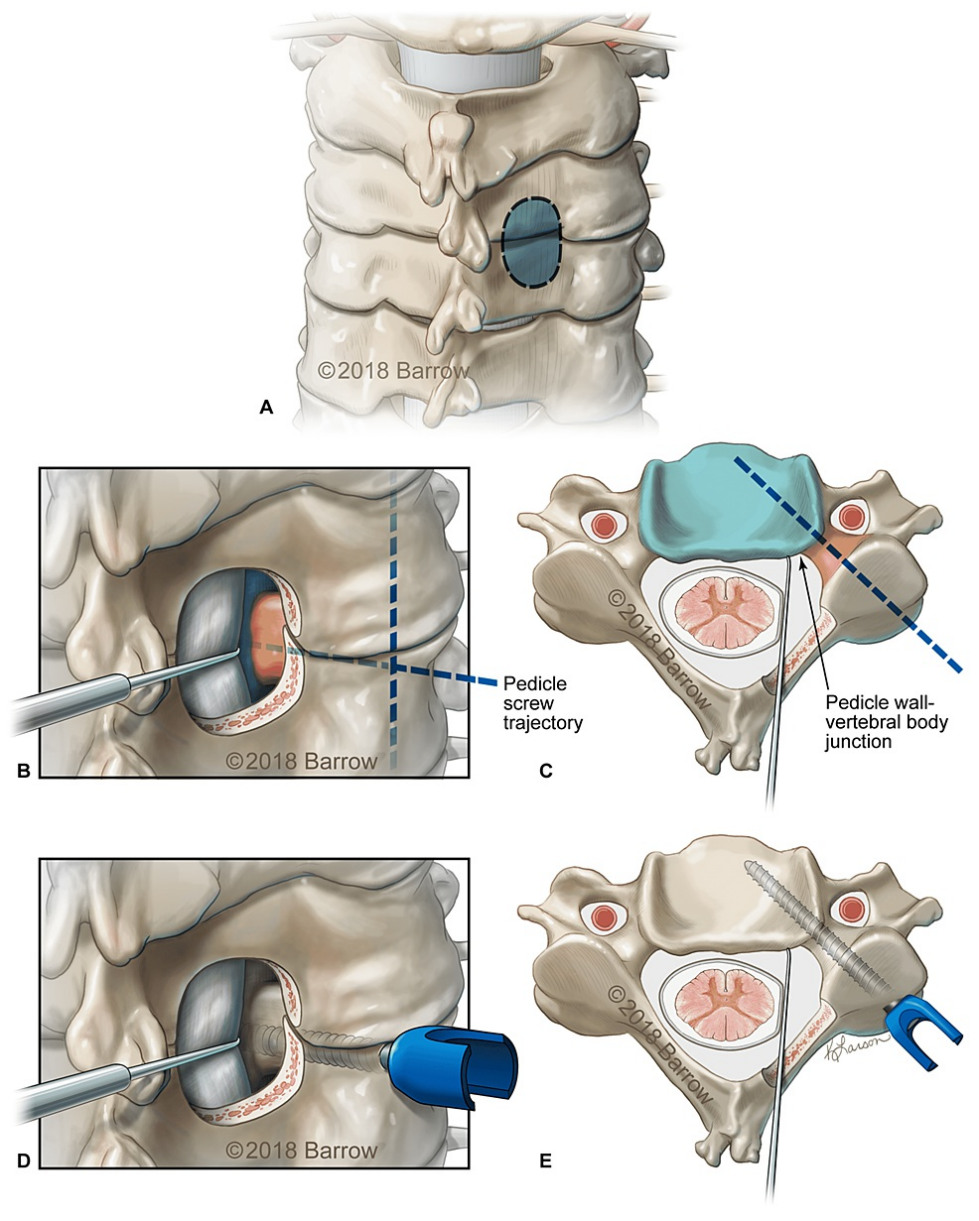
Illustrations showing steps in the freehand technique with direct visualization for subaxial cervical pedicle screw placement. This technique was used for pedicle screw fixation at the lowest instrumented level in occipitocervical fixation and fusion constructs. (A) A hemilaminotomy is performed (dashed line). Posterolateral (B) and axial (C) views showing the superior, inferior, and medial walls of the pedicle. The entry point is placed 1 to 2 mm lateral to the midpoint of the base of the superior articulating process. A dissector is used to retract the dura medially to visualize the interface of the medial pedicle wall with the posterior vertebral body to determine screw trajectory medialization (dashed line). Posterolateral (D) and axial (E) views show proper pedicle screw placement. Used with permission from Barrow Neurological Institute, Phoenix, Arizona.

Numerous techniques for placing subaxial CPSs have been previously reported. The technique we used is a freehand technique that relies on bilateral laminotomies to directly visualize the cervical pedicles during screw insertion (Figure 1).

CPSs were placed only at the lowest instrumented vertebra (LIV). Importantly, the suitability of patient anatomy for the placement of CPSs is confirmed with preoperative computed tomography (CT). Pedicle size and angulation must be appropriate to receive the pedicle screw. First, bilateral hemilaminotomies were performed at the LIV directly medial to the pedicle. The laminotomies were extended laterally and rostral-caudally until the superior, medial, and inferior borders of the pedicle were directly visualized. The surgical assistant aided with visualization by lightly retracting the dura with a Penfield dissector #4. A CPS entry site was then drilled in the lateral third of the LIV’s lateral mass. A small pedicle probe or hand drill was then passed through the pedicle while directly visualizing its trajectory in relation to the pedicle. The trajectory was intentionally over-medialized at first to ensure the avoidance of a vertebral artery injury. Once the hard cortical bone of the medial pedicle wall was encountered via tactile feedback, or after a small medial pedicle breach was directly visualized, the pedicle cannulation trajectory was slightly adjusted laterally to enter the vertebral body. The sagittal plane trajectory is determined by maintaining an orthogonal orientation with the pars. The cannulated pedicle was inspected thoroughly with a ball-tipped probe. A properly sized pedicle screw was then placed under direct visualization of the pedicle. After all fixation anchors had been placed, lateral and anteroposterior fluoroscopic views were obtained to confirm correct hardware placement.

Case 1

**Figure 2:**
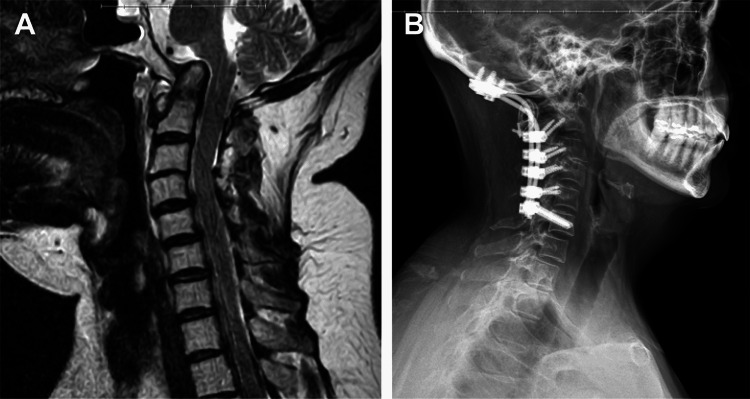
A woman in her early 40s presented with basilar invagination causing cervicomedullary compression in the setting of rheumatoid arthritis. (A) Preoperative sagittal MRI showed cervicomedullary compression. The patient was treated with a fusion from the occiput to C6. Pedicle screws were placed at the distal fixation point at C6 using the described freehand technique with direct visualization. (B) Hardware remained intact on three-year follow-up lateral radiographs. Used with permission from Barrow Neurological Institute, Phoenix, Arizona.

A woman in her early 40s presented with neck pain, myelopathy, radiculopathy, and facial sensory symptoms in the setting of a long history of rheumatoid arthritis. Imaging showed basilar invagination with cervicomedullary compression (Figure 2A).

The patient’s imaging also showed subaxial degeneration with associated listhesis and stenosis (not shown). Surgical correction was recommended given her progressive neurologic deficits and imaging findings. The patient provided consent for an OCF from the occiput to C6. She was placed in traction preoperatively with good reduction of her basilar invagination. Somatosensory and motor evoked potentials were monitored throughout the procedure. Fixation points included an occipital plate, C2 pedicle screws, C3-C5 lateral mass screws, and C6 pedicle screws placed using the described freehand technique with direct visualization. The C6 pedicle screws were 3.5 mm in diameter and 26 mm in length. A fibular strut allograft and cables were used to augment the occipitocervical fixation and arthrodesis. The procedure duration was 225 minutes, with an estimated blood loss of 500 mL. At clinical follow-up seven months after surgery, the patient reported resolution of her preoperative myelopathy and radiculopathy. Postoperative imaging revealed good decompression of the patient’s cervicomedullary junction, good horizontal gaze, and well-placed hardware at all levels. Radiographs at three years showed stable instrumentation and alignment and confirmed arthrodesis with dynamic views (Figure 2B).

Case 2

**Figure 3:**
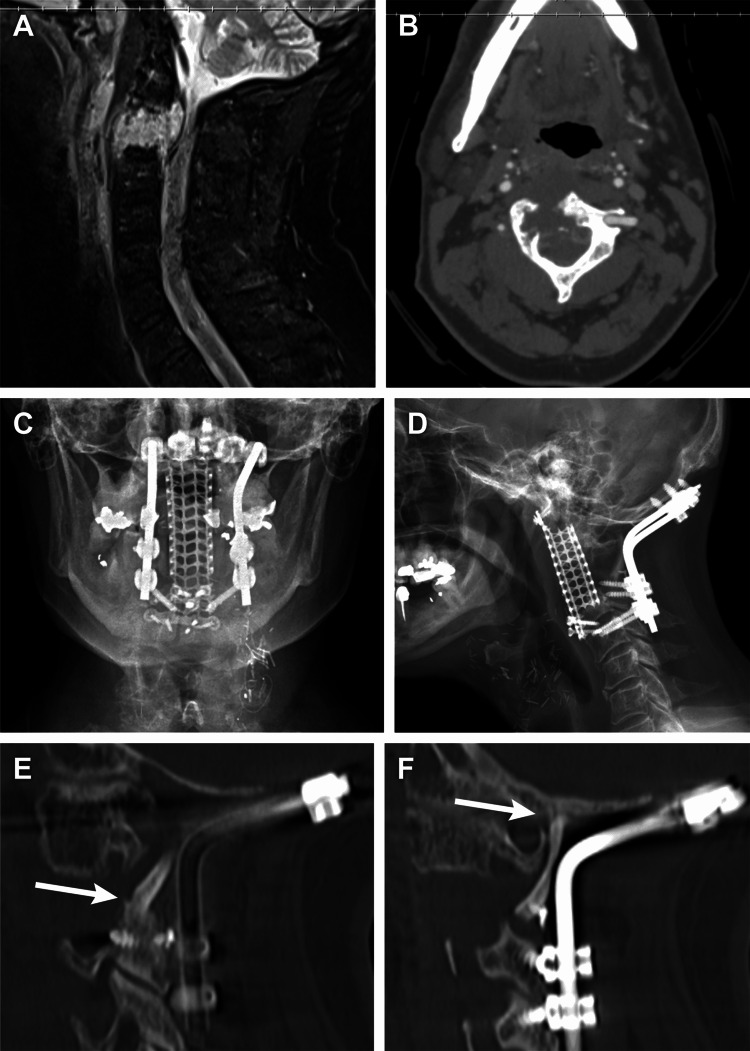
A man in his late 40s presented with a bony lesion at C2 that was found to have enlarged after three years of monitoring. (A) Preoperative sagittal MRI showed the lesion at C2 with epidural extension causing spinal cord compression. (B) Preoperative axial computed tomography (CT) showed a lytic bony lesion eccentric to the right side of C2. A transoral biopsy confirmed a pathologic diagnosis of chordoma, and the patient underwent a two-stage resection and reconstruction surgery. Posterior fixation was performed from the occiput to C4 with pedicle screws placed at the distal anchor point at C4 using the freehand technique with direct visualization. A second-stage anterior approach was then performed for en bloc tumor resection and anterior column reconstruction using a modified Harms cage and screws from the clivus to the C3 vertebral body. Three-month postoperative anterior-posterior (C) and lateral (D) radiographs showed intact hardware. (E-F) Six-month sagittal CT slices showed arthrodesis spanning the corpectomy (arrows). Used with permission from Barrow Neurological Institute, Phoenix, Arizona.

A man in his late 40s presented following a motor vehicle crash. Cervical imaging revealed a bony lesion at C2 and no traumatic injury to the cervical spine. This lesion was initially monitored with routine imaging and was found to have enlarged three years after initial presentation (Figures 3A, 3B).

The patient also developed neck pain with burning symptoms radiating to the back of the head and upper back. A transoral biopsy was performed, and results confirmed a diagnosis of chordoma. Two weeks after the biopsy, a two-stage surgical resection and reconstruction was performed. The first stage was performed posteriorly and included an occiput-C4 fixation with bilateral C4 pedicle screws as the distal fixation point. The C4 pedicle screws were 3.5 mm in diameter and 24 mm in length. The C2 posterior elements were removed, and the vertebral arteries were skeletonized, taking care not to disrupt the tumor. During the exposure, a vascularized occipital bone flap was harvested. This vascularized structural autograft was then secured between the occiput and C2 spinous process using cranial fixation screws to augment the probability of arthrodesis. The next day, the second-stage anterior approach was performed with the assistance of otolaryngology and plastic surgery colleagues. The patient had a tracheostomy placed, followed by mandibulotomy and glossotomy for a transoral approach to C2. The biopsy tract through the pharynx was resected. The vertebral arteries were skeletonized anteriorly from C1 to C3. The anterior arch of C1 was removed, exposing the tip of the dens. The C2-C3 disc space and half of the C3 vertebral body were removed. The chordoma and the remainder of the C2 body were removed en bloc, making every effort to minimize tumor disruption. The anterior column was reconstructed using a modified Harms cage and screws spanning from the clivus to the C3 vertebral body. Plastic surgery and otolaryngology colleagues then reconstructed the pharynx using a radial forearm free flap. The estimated blood loss for both stages combined was 1,400 mL. A gastric tube was placed postoperatively for delivering nutrition while the pharynx healed. One week after surgery, the patient was able to speak and ambulate. He developed a pulmonary embolism on postoperative day 18 and was therapeutically anticoagulated. On postoperative day 22, he was initiated on a regular oral diet and transferred to acute rehabilitation. At the five-month follow-up, the patient reported complete resolution of preoperative neck pain and improvement in myelopathic symptoms. He completed adjuvant proton-beam radiotherapy. His tracheostomy was removed, and his swallowing continued to improve. Postoperative radiographs are shown in Figures 3C, 3D. Six-month CT showed stable instrumentation and confirmed posterolateral arthrodesis spanning the C2 corpectomy (Figures 3E, 3F).

Case 3

**Figure 4:**
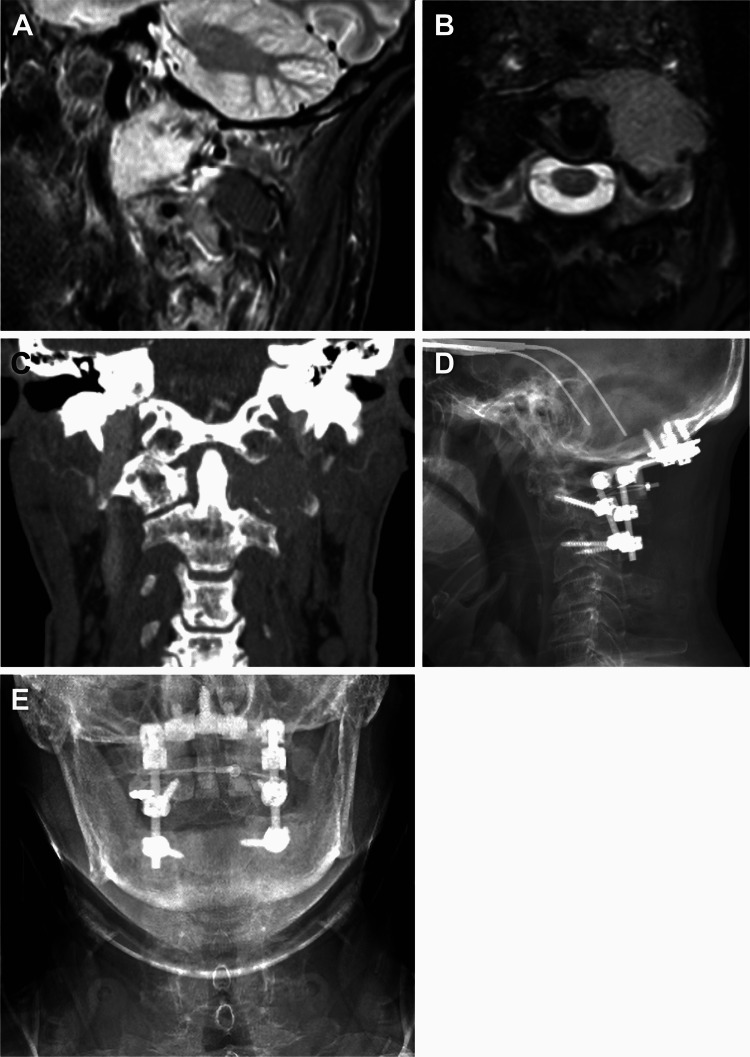
A man in his mid-50s presented with a bony lesion eroding the left C1 lateral mass. Preoperative sagittal (A) and axial (B) MRI showed a lesion in the left C1 lateral mass. (C) Preoperative coronal CT showed the lytic bone lesion. Biopsy results confirmed the diagnosis of plasma cell myeloma. The patient underwent fixation from the occiput to C3 with pedicle screws placed at the distal fixation point at C3 using the freehand technique with direct visualization. Three-month postoperative lateral (D) and anterior (E) radiographs showed intact hardware. Used with permission from Barrow Neurological Institute, Phoenix, Arizona.

A man in his mid-50s presented to the emergency department after a fall with subsequent neck pain and subjective arm weakness. Imaging revealed a bony lesion eroding the left C1 lateral mass, and biopsy results confirmed the diagnosis of plasma cell myeloma (Figures 4A-4C).

The patient initially declined surgical intervention and halo fixation, and thus he was offered a cervical collar. Three months later, he presented to the clinic with worsening neck pain despite collar use. The patient consented to an occiput-C3 fixation and fusion. In the operating room, he was positioned prone with Gardner-Wells tongs and 10 lb of traction. Visual examination and imaging confirmed good neutral alignment and horizontal gaze. Fixation anchors included an occipital plate, one C2 pedicle screw, one C2 pars screw, and bilateral C3 pedicle screws. The C3 pedicle screws were 3.5 mm in diameter and 20 mm in length. A fibular allograft strut was secured from the occiput to the C2 spinous process using cables. The total procedure time was 182 minutes, and estimated blood loss was 300 mL. At the last clinical follow-up five months after surgery, the patient had completed chemotherapy and radiotherapy for his widely metastatic disease. Three-month postoperative radiographs showed intact hardware (Figures 4D, 4E).

## Discussion

Numerous studies have evaluated the biomechanical advantage of CPSs over lateral mass screws, with results indicating a clear advantage for CPSs, especially when under higher pull-out loads, such as those experienced at the LIV of an OCF construct [17,20]. Other studies have reported numerous techniques for placing CPSs, with some claiming high rates of neurovascular injury and many others reporting very few to no incidents of screw-mediated neurovascular injury [9-19].

The primary criticism of CPS placement in the subaxial cervical spine relates to whether a CPS can be placed with the same safety margin as lateral mass screws. Pedicle screw placement in the subaxial spine has been described more commonly at C7; pedicle screw placement in the mid-cervical spine is not as widely established. In our early experience, the direct visualization of the pedicle via laminotomy increases the safety margin of CPS placement to a level that is clinically feasible in the mid-cervical spine. One of the challenges of implementing freehand screw placement techniques for CPS is the variability that exists in cervical pedicle anatomy. By directly visualizing the pedicle during screw placement, one can eliminate this natural anatomic variability as an obstacle to successful CPS placement. Direct visualization, combined with good surgical technique and close monitoring of haptic bony feedback during screw placement, appears to make CPS placement clinically feasible. Care must also be taken with slight retraction of the cervical dura, which involves a marginal risk of cervical spinal cord injury.

However, CPS should not be considered in all patients. We recommended placement of CPS only in patients with adequate-size pedicles and cancellous channels. Patients with small, highly angulated, and sclerotic pedicles are not good candidates for the CPS technique described here. The absence of a cancellous channel within the pedicle reduces the tactile feedback available to the surgeon while cannulating the pedicle. Therefore, the drill is needed to cannulate the pedicle rather than a gear shift probe. Preoperative CT is vital to determine which patients are good candidates for this technique. Adequate size of the pedicles should be confirmed preoperatively. The preoperative CT also serves as an intraoperative reference to assist with screw placement. A learning curve should be expected before this technique can be performed safely.

We recommend that placement of the subaxial pedicle screw at the LIV be performed prior to the screw placement at the adjacent rostral level. The screw tulip head can interfere with the placement of the lateral mass screw and often requires a slightly more rostral entry point for the lateral mass screw. Multiple levels of subaxial pedicle screw placement may also be considered, dependent on suitable patient anatomy.

The current study is limited in that it is a small case series, and future work will require larger case numbers to determine the true safety profile and long-term radiographic and clinical outcomes in these patients. However, this case series supports the feasibility of this technique. The biomechanical advantages of CPS placement should motivate our continued study of this technique, especially for patients who could benefit from a biomechanically more robust distal fixation anchor in the mid-cervical subaxial spine.

## Conclusions

Occipitocervical fixation requires a robust construct with distal fixation anchors able to withstand the loads at the LIV. Pedicle screws are known to have a biomechanical advantage over lateral mass screws and are thus good candidates to serve as distal fixation anchors in these constructs. We report the technical feasibility of and our early clinical experience with the use of subaxial CPS at the LIV of an occipitocervical fusion construct ending in the mid-cervical spine. This freehand technique uses direct visualization of the pedicle to aid in safe and accurate screw placement and is clinically feasible in appropriately selected patients. Future studies with more patients are needed to further validate this technique.
